# Development of a Nomogram for Predicting ICU Readmission

**DOI:** 10.7759/cureus.71555

**Published:** 2024-10-15

**Authors:** Kota Nakano, Junpei Haruna, Ai Harada, Hiroomi Tatsumi

**Affiliations:** 1 Department of Clinical Engineering, Sapporo Medical University Hospital, Sapporo, JPN; 2 Department of Intensive Care Medicine, School of Medicine, Sapporo Medical University, Sapporo, JPN; 3 Department of Nursing, Sapporo Medical University Hospital, Sapporo, JPN

**Keywords:** icu readmission, intensive care unit, nomogram, prediction model, sleep disturbance

## Abstract

Background

This study aims to develop and validate a comprehensive prediction model for ICU readmissions. Readmission following ICU discharge is associated with adverse outcomes such as increased mortality, prolonged hospital stays, and elevated healthcare costs. Consequently, predicting and preventing readmissions is crucial. Previous models for predicting ICU readmissions were primarily based on physiological indices; however, these indices fail to capture the complete nature of treatment or patient conditions beyond physiological measures, thereby limiting the accuracy of these predictions.

Methodology

A total of 1,400 patients who had an unplanned ICU admission at Sapporo Medical University Hospital from January 2015 to October 2022 were included; a single regression analysis was performed using unplanned ICU readmission as the dependent variable. After performing a single regression analysis, logistic regression analysis using the stepwise method was performed using variables with significant differences, and a predictive nomogram was created using the variables that remained in the final model. To internally validate the predictive nomogram model, nonparametric bootstrapping (1,000 replications) was performed on the original model.

Results

Of the 1,400 patients who had an unplanned admission to the ICU, 114 (8.1%) were readmitted to the ICU unplanned. Seven main variables (Sequential Organ Failure Assessment score, respiratory rate, Glasgow Coma Scale, sleep disturbance, Continuous Kidney Replacement Therapy, presence of tracheal suctioning, and Oxygen Saturation) were selected to be associated with ICU readmission. The evaluation of the models showed excellent discrimination with an area under the receiver operating characteristic of 0.805 (original model) and 0.796 (bootstrap model). Calibration plots also confirmed good agreement between observed and predicted reentry.

Conclusions

This new predictive model is more accurate than previous models because it includes physiological indicators as well as other patient conditions and procedures needed and is expected to be used in clinical practice. In particular, the inclusion of new factors, such as sleep disturbance and the need for tracheal suctioning, enabled a more comprehensive patient assessment. The use of this predictive nomogram as a criterion for discharging ICU patients may prevent unplanned ICU readmission.

## Introduction

Unplanned intensive care unit (ICU) readmissions occur in 4.5% to 9.2% of patients discharged from the ICU and are events that must be prevented because of their negative impact on patient outcomes and the hospital as a whole [1]. Unplanned readmission to ICUs not only increases mortality (21.4%-40%) in patient outcomes but also negatively impacts hospital outcomes, including prolonged length of stay [2] and increased healthcare costs [3]. However, methods to accurately predict ICU readmissions have yet to be established.

Physiological measures such as the Stability and Workload Index for Transfer [4] and the National Early Warning Score (NEWS) [5] at ICU discharge have been used to predict unplanned ICU readmission. Nonetheless, these measures have limited validity because they do not reflect the nature of ICU treatment or patient factors such as delirium and sleep. In addition, the factors associated with ICU readmission are diverse and difficult to predict with a single measure or factor.

Therefore, it would be clinically significant to develop a prediction model for ICU readmission that uses comprehensive data on the patient's physical and mental status and the procedures required for the patient, in addition to the nature of treatment during ICU and vital signs at discharge. This study aims to develop and validate a comprehensive prediction model for ICU readmissions.

## Materials and methods

Design, setting, and inclusion criteria

More data are required to develop a comprehensive nomogram for predicting ICU readmission. Therefore, we conducted a single-center retrospective observational study. The study design and protocol were approved by the Institutional Review Board (IRB) of Sapporo Medical University (IRB approval number 342-157, December 1, 2022). Owing to the observational nature of this study, the information was released on an opt-out basis. In this study, we used information from previous ICU patients, so it was difficult to obtain consent directly from the patients. Therefore, we published the purpose of this study on a web page within the research group and provided each patient with the opportunity to refuse to participate in the study. Data was collected from the electronic health records of patients who were admitted to the ICU at Sapporo Medical University Hospital for unplanned reasons between January 2015 and October 2022. In this study, we made sure to sufficiently reduce the bias in patient information that can occur in retrospective observational studies through statistical processing. These patients, excluding those meeting exclusion criteria, were enrolled; the three researchers collected data using a dedicated data sheet between January and March 2023. A third party checked for any inconsistencies in the data. Based on previous studies, ICU readmission was defined as readmission to the ICU within seven days after ICU discharge [6,7].

Exclusion criteria

We did not collect data on patients who fell under the following categories: (1) patients under 18 years of age, because pediatric patients require specialized care due to differences in physiology, treatment protocols, and equipment needs, (2) patients who were deceased in the ICU, (3) patients who had signed DNAR consent forms when leaving the ICU as these patients would not be readmitted to the ICU even if their condition worsens, (4) patients who were transferred to another hospital within seven days following ICU discharge, and (5) patients who were lacking vital signs or other essential items at the time of ICU discharge.

Data collection

We collected the most recent patient data from our electronic medical record within 24 hours before ICU discharge. Patient characteristics such as age, sex, and underlying medical conditions were collected, and data on the Charlson Comorbidity Index, ICU length of stay, mortality rate, presence of sepsis, Acute Physiology and Chronic Health Evaluation (APACHE) II score at ICU, Sequential Organ Failure Assessment (SOFA) score, mechanical ventilation, mechanical ventilator days, continuous kidney replacement therapy (CKRT), intra-aortic balloon pumping, Impella®︎, extracorporeal membrane oxygenation, and high-flow nasal cannula oxygen use were also collected. The results of blood gas analysis at ICU discharge were collected for PaO_2_, PaCO_2_, and pH. Vital signs collected at ICU discharge included respiratory rate (RR), temperature (BT), systolic blood pressure, diastolic blood pressure, heart rate (HR), cold extremities, tracheal suctioning, sleep disturbance, and delirium. Vital signs (RR, BT, SpO_2_) were selected because they are frequently used as early warning signs and are versatile parameters that are generally measured in clinical practice.

Sleep data were collected on the day before the patient was discharged from the ICU, whether or not they had a sleep disturbance. Although there was no sleep assessment tool, the sleep status of ICU patients was recorded in the electronic medical record every day at the research facility. Sleep disorders were determined to be present when there was a subjective complaint of insomnia from the patient or when the patient was judged to have insomnia based on information from the nurse's observations of sleep [8]. Delirium was also assessed using the intensive care delirium screening checklist score on the day before discharge from the ICU, and a score of 4 or higher was defined as delirium [9].

Criteria for ICU discharge

The intensivists, attending physicians, and ICU medical staff shared the patient's condition through morning rounds and meetings to determine if the patient was suitable for ICU discharge according to the ICU admission, discharge, and triage guidelines [10]. Communication was based on the ICU admission, discharge, and triage guidelines, and the final decision to discharge the patient from the ICU was made after a consensus was reached by the team. The ICU discharge criteria for our ICU were as follows: (1) stable patient not requiring ICU treatment or monitoring. (2) In a multidisciplinary setting with the attending physician, it was confirmed that the patient was ready for ICU discharge.

Statistical analysis

Data were evaluated for Gaussian distribution using the Shapiro-Wilk normality test. Normally distributed data were presented as mean ± standard deviation (SD), and non-normally distributed data were presented as median and interquartile range (IQR). Because the number of ICU readmission cases in this study was small, we used the Shapiro-Wilk test to check for normality. Categorical data were presented as counts and corresponding frequencies (%). Patient characteristics were analyzed using the Mann-Whitney U test for continuous variables and Fisher's exact test for categorical variables.

Univariate and single regression analyses were performed with ICU readmission as the dependent variable to establish an appropriately calibrated nomogram to predict outcomes. Logistic regression analysis using a stepwise decreasing method was performed using factors with *P*-values less than 0.05 as a result of the single regression analysis. In the logistic regression analysis, we set the significance level at 0.05. Predictive nomograms were created using the remaining variables in the final model of the logistic regression analysis. Nonparametric bootstrapping (1,000 replications) of the original model was performed for internal validation of the model for the predictive nomogram [11]. Using the bootstrap method, the bias of the model can be quantitively evaluated using resampled data and its impact can be reduced. Furthermore, training the model on various subsets of the data may improve the model's prediction accuracy. This is because it is expected to reduce the risk of overfitting and improve the model's ability to make predictions on new data. By creating models for multiple data sets using bootstrapping, the adaptability of the model to new data (generalization ability) can be evaluated. By using a variety of data sets through resampling, the risk of the model becoming overly specific to a particular data set can be reduced, and an indicator for creating a model with high generalization performance can be obtained. The bootstrap sample was created by extracting random samples from the development database. To confirm the identification, AUROCs obtained from the development database and bootstrap were calculated, respectively. As a validation, overfitting was checked based on optimism <0.2, and a calibration plot was created [12]. Based on these results, a nomogram prediction model based on independent risk factors associated with ICU readmission was constructed. The significance level was set at less than 5%. The analysis software used for this study was R version 4.2.2 (The R Foundation for Statistical Computing, Vienna, Austria). The R package rms was used to create the nomogram.

## Results

Patient characteristics

**Figure 1:**
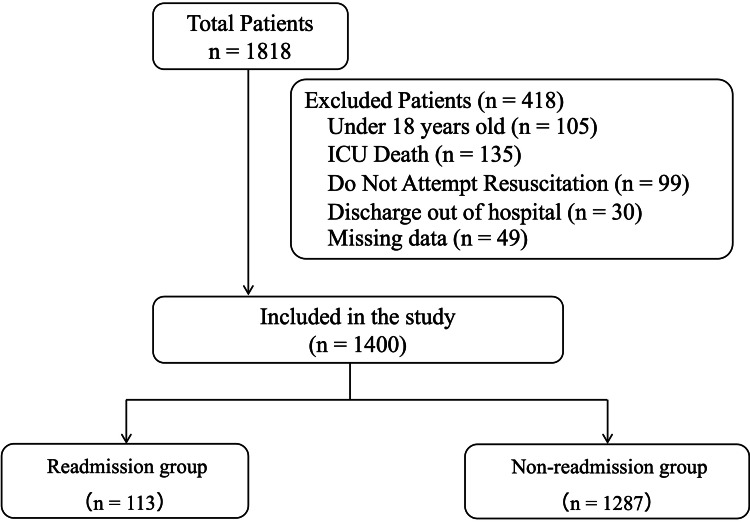
Patient enrollment flowchart.

Figure 1 shows the flowchart of patient enrollment. Of the 1,818 patients who had unplanned ICU admission during the study period, 418 were excluded based on exclusion criteria. The remaining 1,400 patients were included in the study. A total of 113 patients (8.1%) were readmitted to the ICU within seven days of ICU discharge.

Patient characteristics are shown in Table 1. Compared with the ICU non-readmission group, the ICU readmission group had significantly higher APACHE II and SOFA scores at the time of ICU admission (18 vs. 19, *P* = 0.002; 6 vs. 7, *P *< 0.001). In addition, the ICU readmission group had significantly higher HR, RR, and BT at ICU discharge than the ICU non-readmission group (69 vs. 69, *P *< 0.001; 21 vs. 19, *P *< 0.001; 37.0 vs. 37.2; *P* = 0.006). Furthermore, the SpO_2_ and GCS at ICU discharge were significantly lower in the ICU readmission group than in the ICU non-readmission group (97 vs. 98; *P* = 0.003). Patients who required tracheal suctioning for expectoration and those who had sleep disorders were significantly more common in the ICU readmission group (30, 26.5%, vs. 154, 12.0%, *P *< 0.001; 72, 63.7%, vs. 193, 15%, *P *< 0.001). The ICU mortality rate was significantly higher in the ICU readmission group (11, 9.7%, vs. 54, 4.2%, *P* = 0.014).

**Table 1:** Patient characteristics. APACHE Ⅱ, Acute Physiology and Chronic Health Evaluation Ⅱ; SOFA, Sequential Organ Failure Assessment; CKRT, Continuous Kidney Replacement Therapy; PMX-DHP, direct hemoperfusion using polymyxin B-immobilized fiber column; IABP, Intra-Aortic Balloon Pumping; Impella®️, percutaneous ventricular assist device; SBP, systolic blood pressure; DBP, diastolic blood pressure; HR, heart rate; BT, body temperature; RR, respiratory rate; SpO_2_, saturation of percutaneous oxygen; GCS, Glasgow Coma Scale; P/F, partial pressure of arterial oxygen/fraction of inspiratory oxygen ratio; PaCO_2_, partial pressure of arterial carbon dioxide; HFNC, high-flow nasal cannula

-	Overall (*n* = 1,400)	Readmission group (*n* = 113)	Non-readmission group (*n* = 1,287)	*P*-value
Demographic data				
Age (years), median (IQR)	68 (56-76）	70 (59-77）	68 (56-76)	0.318
Male, *n* (%)	836 (59.7)	74 (65.5)	762 (59.2)	0.238
Clinical characteristics				
Charlson Comorbidity Index, median (IQR)	5 (3-6)	5 (3-7)	5 (3-6)	0.460
APACHE II, median (IQR)	18 (15-22)	19 (16-23)	18 (15-21)	0.002
SOFA at ICU admission, median (IQR)	6 (4-8)	7 (5-10)	6 (4-8)	<0.001
ICU length of stay (days), median (IQR)	3 (2-6)	4 (2-8)	3 (2-6)	0.002
Mortality for 28 days	65 (4.6)	11 (9.7)	54 (4.2)	0.014
ICU admission source				
Medical, *n* (%)	797 (56.9)	79 (69.9)	718 (55.8)	0.005
Surgical, *n* (%)	603 (43.1)	34 (30.1)	569 (54.2)	0.005
Reasons for ICU admission				
Sepsis, *n* (%)	256 (18.3)	28 (24.8)	228 (17.2)	0.082
Cardiovascular surgery, *n* (%)	195 (13.9)	13 (11.5)	182 (14.1)	0.526
Other surgery, *n* (%)	405 (28.9)	21 (18.6)	384 (29.8)	0.015
Respiratory failure, *n* (%)	220 (15.7)	29 (25.7)	191 (14.8)	0.004
Circulatory failure, *n* (%)	145 (10.4)	11 (9.7)	134 (10.4)	0.948
Cerebrovascular disease, *n* (%)	24 (1.7)	1 (0.9)	23 (1.8)	0.741
Acute kidney injury, *n* (%)	59 (4.2)	1 (0.9)	58 (4.5)	0.111
Acute pancreatitis, *n* (%)	31 (2.2)	4 (3.5)	27 (2.1)	0.506
Endocrine disease, *n* (%)	67 (4.8)	3 (2.7)	64 (5.0)	0.381
Liver failure, *n* (%)	27 (1.9)	5 (4.4)	22 (1.7)	0.097
Metabolic disorder, *n* (%)	16 (1.1)	1 (0.9)	15 (1.2)	1.000
Treatment variables of the group				
Mechanical ventilation, *n* (%)	757 (54.1)	65 (57.5)	692 (53.8)	0.503
Ventilator days, median (IQR)	1.0 (0-3.0）	2.0 (0-5.3）	1.0 (0-3.0）	0.006
CKRT, *n* (%)	253 (18.1)	36 (31.9)	217 (16.9)	<0.001
PMX-DHP, *n* (%)	74 (5.3)	10 (8.8)	64 (5.0)	0.122
IABP, *n* (%)	30 (2.1)	0 (0)	30 (2.3)	0.193
Impella®️, *n* (%)	7 (0.5)	1 (0.9)	6 (0.5)	1.000
Tracheal suctioning, *n* (%)	184 (13.1)	30 (26.5)	154 (12.0)	<0.001
Use of catecholamine, *n* (%)	51 (3.6)	5 (4.4)	46 (3.5)	0.841
Low-flow oxygen therapy, *n* (%)	946 (67.6)	82 (72.6)	864 (67.1)	0.281
HFNC, *n* (%)	86 (6.1)	3 (2.7)	83 (6.5)	0.160
Parameters at ICU discharge				
SBP (mmHg), median (IQR)	121 (108-135)	123 (108-139)	121 (108-135)	0.457
DBP (mmHg), median (IQR)	69 (60-79)	69 (60-83)	69 (60-78)	0.906
HR (bpm), median (IQR)	83 (72-96)	89 (80-102)	82 (72-95)	<0.001
BT (℃), median (IQR)	37.0 (37.0-37.8)	37.2 (37.0-38.0)	37.0 (36.9-37.7)	0.006
RR (bpm), median (IQR)	19 (16-22)	21 (17-25)	19 (16-22)	<0.001
SpO_2 _(%), median (IQR)	98 (96-99)	97 (96-98)	98 (96-99)	0.003
GCS, median (IQR)	15 (14-15)	14 (12-15)	15 (14-15)	<0.001
Cold extremities, *n* (%)	124 (8.9)	37 (32.7)	87 (6.8)	<0.001
P/F, median (IQR)	357 (269-438)	333 (238-397)	358 (274-443)	0.004
PaCO_2 _(mmHg), median (IQR)	37.0 (34.0-41.0)	36.0 (34.2-41.3)	37.0 (34.0-41.0)	0.514
pH, median (IQR)	7.43 (7.40-7.47)	7.44 (7.41-7.48)	7.43 (7.40-7.47)	0.059
Sleep disturbance, *n* (%)	265 (18.9)	72 (63.7)	193 (15.0)	<0.001
Delirium, *n* (%)	116 (8.3)	11 (9.7)	91 (7.1)	0.342
Reason for ICU readmission				
Respiratory failure, *n *(%)	-	46 (40.7)	-	-
Circulatory failure, *n* (%)	-	35 (31.0)	-	-
Renal failure, *n* (%)	-	13 (11.5)	-	-
Electrolyte disturbances, *n* (%)	-	10 (8.8)	-	-
Neurological failure, *n* (%)	-	9 (8.0)	-	-

Predictors of ICU readmission

The results of a logistic regression analysis (stepwise decreasing method) to explore predictors of ICU readmission are shown in Table 2.

**Table 2:** Results of univariate and multivariate analysis. APACHE Ⅱ, Acute Physiology and Chronic Health Evaluation Ⅱ; CCI, Charlson Comorbidity Index; SOFA, Sequential Organ Failure Assessment; CKRT, Continuous Kidney Replacement Therapy; PMX-DHP, direct hemoperfusion using polymyxin B-immobilized fiber column; IABP, Intra-Aortic Balloon Pumping; Impella®️, percutaneous ventricular assist device; SBP, Systolic Blood Pressure; DBP, diastolic blood pressure; HR, heart rate; BT, body temperature; RR, respiratory rate; SpO_2_, saturation of percutaneous oxygen; GCS, Glasgow Coma Scale; P/F, partial pressure of arterial oxygen/fraction of inspiratory oxygen; PaCO_2_, partial pressure of arterial carbon dioxide; HFNC, high-flow nasal cannula

Variables	Unadjusted		Adjusted	
	Odds ratio (95% CI)	*P*-value	Odds ratio (95% CI)	*P*-value
Age	1.006 (0.994-1.022)	0.326		
Sex	1.301 (0.997-1.947)	0.202		
CCI	1.019 (0.948-1.095)	0.617		
APACHE Ⅱ	1.050 (1.019-1.081)	0.001		
SOFA	1.124 (1.063-1.188)	<0.001	1.078 (1.015-1.145)	0.014
Sepsis	1.530 (0.975-2.401)	0.064		
ICU LOS	1.019 (0.952-1.090)	0.410		
MV	1.164 (0.789-1.718)	0.443		
MV days	1.053 (1.017-1.091)	0.980		
Tracheotomy	1.342 (0.780-2.311)	0.288		
PMX-DHP	1.855 (0.925-3.722)	0.082		
CKRT	2.305 (1.512-3.515)	<0.001	1.769 (1.122-2.790)	0.014
ECMO	0.629 (0.083-4.759)	0.654		
IABP	0 (0-0)	0.998		
Impella^®︎^	1.906 (0.227-15.97)	0.552		
SBP	1.004 (0.995-1.014)	0.360		
DBP	1.002 (0.998-1.016)	0.768		
HR	1.020 (1.010-1.031)	<0.001		
BT	1.422 (1.091-1.855)	0.009		
RR	1.094 (1.057-1.132)	<0.001	1.072 (1.034-1.122)	<0.001
SpO_2_	0.915 (0.842-0.995)	0.037	0.922 (0.854-1.005)	0.066
GCS	0.877 (0.817-0.941)	<0.001	0.863 (0.794-0.938)	<0.001
P/F	0.998 (0.996-0.999)	0.006		
PaCO_2_	1.000 (0.999-1.002)	0.684		
pH	11.68 (0.403-338.4)	0.154		
Sleep disorder	9.954 (6.587-15.04)	<0.001	9.742 (6.315-15.02)	<0.001
Delirium	1.417 (0.734-2.735)	0.298		
Tracheal suctioning	2.659 (1.695-4.171)	<0.001	2.053 (1.288-3.273)	0.002
Catecholamine	1.249 (0.486-3.209)	0.644		
Cold extremities	1.094 (0.518-2.310)	0.814		
Low-flow oxygen therapy	0.764 (0.513-1.136)	0.184		
HFNC	0.846 (0.361-1.985)	0.701		

Logistic regression analysis was performed, including variables that were significantly different from the univariate analysis and clinically necessary variables that may be associated with ICU readmission. These included APACHE II, SOFA, sepsis, ventilatory duration, PMX, CKRT, HR, BT, RR, SpO_2_, tracheal suctioning, sleep disturbance, GCS, P/F, and PaO_2_. 

**Figure 2:**
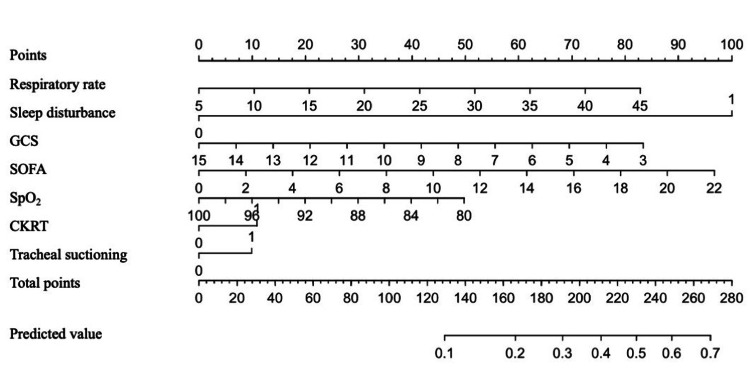
Nomogram for predicting ICU readmission. A nomogram for predicting ICU readmission based on seven easily obtainable clinical characteristics. To use the nomogram, place the patient's variables on the corresponding axes, draw a line on the point axis, add up the points, and draw a line from the total point axis to the predicted value. SOFA, Sequential Organ Failure Assessment; CKRT, Continuous Kidney Replacement Therapy; SpO_2_, saturation of percutaneous oxygen; GCS, Glasgow Coma Scale

We created a nomogram for each of the seven variables obtained as the final model using logistic regression analysis (stepwise method) (Figure 2). These included respiratory rate, sleep disorder, GCS, SOFA, SpO_2_, tracheal suctioning, and CKRT.

Evaluation of the models

**Figure 3:**
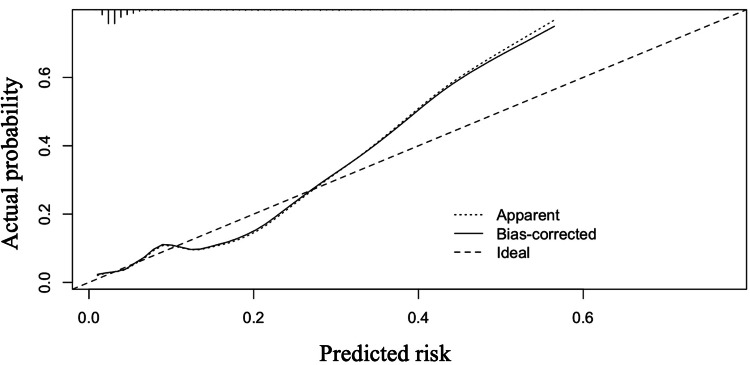
Illustrations of miscalibration. Illustrations are a model with an area under the receiver operating characteristic curve (AUROC or c-statistic) of 0.80. Calibration intercept and slope are indicated for each illustrative curve.

The area under the receiver operating characteristic (AUROC) curve for the predicted risk of ICU readmission was 0.805 and 0.796 for the original and bootstrap models, respectively, showing very good discrimination. The validation of overfitting obtained with the bootstrap confirmed that the data in this study were within the overfitting criterion, with an optimism of 0.038. The calibration plot (Figure 3) showed good agreement between observed and predicted ICU reentry. The figure shows a model with an AUROC curve (c-statistic) of 0.80. Each of the illustrated curves shows the calibration intercept and slope. For example, when the actual probability on the y-axis is approximately 50%, the predicted risk on the x-axis is approximately 40%, indicating that there is a possibility of ICU readmissions being underestimated. Therefore, it is recommended to use the prediction nomogram in this study, taking this consideration into account.

## Discussion

Patients readmitted to the ICU are more severely ill, resulting in longer hospital stays and increased costs [1]. Some reports indicate that 26%-58% of patients readmitted to the ICU die [13]. Establishing an effective ICU readmission prediction model has been shown to minimize readmissions [14]. Vital signs have been included as early warning signs to predict the prognosis of critically ill patients. Consistent with previous studies, they are considered to be important variables for predicting ICU readmission. Most studies that predict ICU readmission consider only blood data or vital signs and do not include the nature of treatment during ICU, the patient's mental status, or the type of procedure the patient needs [15]. In our study, we successfully developed a predictive nomogram model to identify the likelihood of ICU readmission, including the treatment factors and physiological parameters during ICU hospitalization, as well as the patient's required procedures, patient's delirium, and sleep status.

Our study included comprehensive data on critical illness scores, vital signs, and ICU treatment details. The variables we included are consistent with those of other studies [14,16,17]. Furthermore, the SOFA score is a risk factor that is often incorporated into ICU quality outcomes as well as predictive models for ICU readmission [18,19]. Additionally, it includes respiratory system parameters such as respiratory rate and SpO_2_. Respiratory rate has been widely reported as a parameter that can be used before a patient becomes critically ill; it is also included in early warning scores, including the NEWS [20], and is a parameter that acutely reflects a patient's physical condition [21].

Some papers incorporate the presence or absence of renal replacement therapy as part of ICU treatment details [22]. Moreover, CKRT may be used to remove cytokines in patients with sepsis or to assist with kidney function [23]. These treatments are designed to correct organ damage, and the use of CKRT necessarily implies a higher degree of severity of disease.

Unlike other models predicting ICU readmission, procedures related to tracheal suctioning were included as a factor predicting readmission in this study. Notably, the presence and frequency of suctioning are considered an important factor in patient airway management, as the frequency of tracheal suctioning is associated with reintubation [24]. Clearing the airway is also an important factor in preventing ICU readmission, as many patients are readmitted with respiratory failure.

Sleep disturbance is one variable that is often overlooked in the ICU. ICU patients are often given analgesic and sedative drugs, and their sleep is often not assessed. It is well known that sleep disorders are associated with patient delirium [25]. Furthermore, sleep is important for the prevention of post-intensive care syndrome and the promotion of physical activity [26]. Although ICU admission delirium is not included in this model, it may lead to the development of post-ICU discharge delirium and hinder physical recovery. Daily sleep assessment of ICU patients may be able to predict ICU readmission. Sleep disturbances are another factor that differs from other predictors of ICU readmission.

Strengths and limitations

The strength of this study is that the predictive model includes not only vital signs but also patient sleep patterns and required procedures. It may be possible to predict the risk of ICU readmission for patients with sleep disorders by evaluating sleep during ICU admission. The AUROC results of the predictive nomogram obtained in our study were comparable to those of previous studies [15]. On the other hand, this study has several limitations. First, because the data in this study was obtained retrospectively from a single facility, it is possible that there was not enough data to establish a prediction model, and there may be limitations to the generalization of the results. Future development of a prediction model using multicenter data or other available databases may be necessary. Second, the limited amount of data in this study made external validation difficult. However, to compensate for this, we performed validation using the bootstrap method. The bootstrap method involves repeatedly randomly sampling from the original data, constructing multiple models based on these samples, and then aggregating and evaluating the results. The main benefit of this method is that it enables the estimation of unknown errors and fluctuations in the original data by resampling. We believe that using this method has helped minimize bias. Third, we obtained metabolic and respiratory data via blood gas analysis; however, we did not collect variables such as those related to nutritional status or infection. The inclusion of these variables could improve the efficiency of the prediction model. Fourth, this prediction model was not prospectively validated. We plan to conduct a multicenter prospective study of this ICU readmission prediction model.

Implications for clinical practice

The ICU readmission prediction model developed by this study has important implications for clinical practice. The model, which incorporates patient sleep patterns and patient care needs in addition to common vital signs, has the potential to improve the accuracy of assessing readmission risk compared to traditional prediction models. In particular, the inclusion of factors that are often overlooked in conventional models, such as sleep disturbances and the need for tracheal suctioning, allows for a more comprehensive patient assessment. In the future, it will be necessary to investigate the impact of implementing the predictive model obtained in this study in clinical practice and to evaluate its effectiveness in various patient populations.

## Conclusions

The current study developed and demonstrated the utility of a comprehensive model for predicting unplanned readmission after ICU discharge. The model, which takes into account the patient's sleep status and treatment, needs in addition to physiological indicators, contributes to an accurate assessment of readmission risk and allows for appropriate interventions for healthcare professionals. Furthermore, it is thought to be involved in the optimization of the allocation of medical resources and patient safety. The use of this model is expected to improve the accuracy of decisions regarding the timing of ICU discharge, leading to improved patient outcomes and safety, as well as the appropriate allocation of medical resources. In the future, it will be necessary to strengthen the evidence for the predictive model by accumulating more data and conducting external validation.
